# Petunia PHYTOCHROME INTERACTING FACTOR 4/5 transcriptionally activates key regulators of floral scent

**DOI:** 10.1007/s11103-024-01455-8

**Published:** 2024-05-30

**Authors:** Ekaterina Shor, Alexander Vainstein

**Affiliations:** 1 Institute of Plant Sciences, ARO, Volcani Institute, Rishon Lezion, Israel; 2 The Robert H. Smith Institute of Plant Sciences and Genetics in Agriculture, The Hebrew University, Rehovot, Israel

**Keywords:** Floral scent, Regulation, PIF4, EOBII, DELLA

## Abstract

Floral scent emission of petunia flowers is regulated by light conditions, circadian rhythms, ambient temperature and the phytohormones GA and ethylene, but the mechanisms underlying sensitivity to these factors remain obscure. PHYTOCHROME INTERACTING FACTORs (PIFs) have been well studied as components of the regulatory machinery for numerous physiological processes. Acting redundantly, they serve as transmitters of light, circadian, metabolic, thermal and hormonal signals. Here we identified and characterized the phylogenetics of petunia PIF family members (PhPIFs). PhPIF4/5 was revealed as a positive regulator of floral scent: TRV-based transient suppression of *PhPIF4/5* in petunia petals reduced emission of volatiles, whereas transient overexpression increased scent emission. The mechanism of PhPIF4/5-mediated regulation of volatile production includes activation of the expression of genes encoding biosynthetic enzymes and a key positive regulator of the pathway, EMISSION OF BENZENOIDS II (EOBII). The PIF-binding motif on the *EOBII* promoter (G-box) was shown to be needed for this activation. As PhPIF4/5 homologues are sensors of dawn and expression of *EOBII* also peaks at dawn, the prior is proposed to be part of the diurnal control of the volatile biosynthetic machinery. PhPIF4/5 was also found to transcriptionally activate *PhDELLA*s; a similar positive effect of PIFs on *DELLA* expression was further confirmed in *Arabidopsis* seedlings. The PhPIF4/5–PhDELLAs feedback is proposed to fine-tune GA signaling for regulation of floral scent production.

**Supplementary Information:**

The online version contains supplementary material available at 10.1007/s11103-024-01455-8.

## Key message

Petunia homolog of *Arabidopsis* PIF4 and PIF5 (PhPIF4/5) regulates specialized metabolism in flower corolla and is involved in linking of partially independent routes leading to regulation of floral scent production.

**Supplementary Information:**

The online version contains supplementary material available at 10.1007/s11103-024-01455-8.

## Introduction

*Petunia* x *hybrida* is a model plant for floral scent and petal pigmentation studies (Quattrocchio et al. 1999; Verdonk et al. 2003). Petunia flowers emit a number of phenylalanine (Phe)-derived volatile organic compounds (VOCs): methyl benzoate, benzaldehyde, benzyl benzoate, benzyl alcohol, benzyl acetate (C6-C1 derivatives); phenylethyl alcohol, phenylethyl acetate and benzeneacetaldehyde, with a C6-C2 carbon skeleton; eugenol and isoeugenol with a C6-C3 structure (Muhlemann et al. 2014; Skaliter et al. 2022). The enzymes catalyzing the major steps of VOC biosynthesis have been identified and characterized, among them 5-enolpyruvylshikimate-3-phosphate synthase (EPSPS) and arogenate dehydratase (ADT), both involved in phenylalanine biosynthesis; L-phenylalanine ammonia lyase (PAL) and phenylacetaldehyde synthase (PAAS), catalyzing the first reactions of the two main branches of the phenylpropanoid pathway leading to the production of C6-C1/C6-C3 and C6-C2 volatiles, respectively; S-adenosyl-L-methionine: benzoic acid/salicylic acid carboxyl methyltransferase (BSMT), benzoyl-CoA: benzyl alcohol/2-phenylethanol benzoyltransferase (BPBT), and isoeugenol synthase (IGS) involved in later steps of VOC biosynthesis (Maeda and Dudareva 2012; Dudareva et al. 2013).

Floral VOC production is controlled at different levels. Transcriptional activation of biosynthetic genes includes MYB-transcription factors: ODORANT1 (ODO1) and EMISSION OF BENZENOIDS (EOB) I, and EOBII. The latter acts upstream of EOBI and ODO1 (Verdonk et al. 2005; Spitzer-Rimon et al. 2010, 2012; Colquhoun et al. 2011). GRAS protein PHENYLPROPANOID EMISSION-REGULATING SCARECROW-LIKE (PES) has also been shown to enhance scent via transcriptional activation of scent-related genes (Shor et al. 2023a). The recently identified repressor of scent UNIQUE PLANT PHENYLPROPANOID REGULATOR (UPPER) has been proposed to act at the post-transcriptional level (Shor et al. 2023b). In addition, VOC biosynthesis is determined by Phe availability which, in turn, is controlled by feedback of both its level and the levels of synthesized VOCs (Lynch and Dudareva 2020; Liao et al. 2021). VOC emission is also influenced by the genes facilitating their release into the environment (Cna’ani et al. 2015b; Adebesin et al. 2017; Liao et al. 2021, 2023).

Volatile production in *Petunia* x *hybrida* is diurnally governed and is increased at night to attract its nocturnal pollinators (Fenske et al. 2018). Diurnal control of physiological processes usually involves both circadian and light signaling (Dodd et al. 2005; Nozue et al. 2007; Inoue et al. 2017). Scent emission is regulated by the circadian clock components LATE ELONGATED HYPOCOTYL and GIGANTEA (Fenske et al. 2015; Brandoli et al. 2020). The effect of light on scent production has not been studied in depth. Emission of the major VOCs by petunia flowers is higher under constant light than constant darkness (Fenske et al. 2015; Shor et al. 2023a). Moreover, light quality affects VOC biosynthesis. Far-red light activates floral scent via the light photoreceptor PHYTOCHROME A (PHYA) (Shor et al. 2023a). Red and blue light spectral ranges also impact the regulation of VOC levels (Colquhoun et al. 2013); however, the molecular mechanisms underlying this regulation remain obscure.

Physiological responses to far-red and red light are regulated by the basic helix–loop–helix (bHLH) transcription factors termed PHYTOCHROME INTERACTING FACTORs (PIFs), which physically interact with photoreceptors (Pedmale et al. 2016; Pham et al. 2018). PIFs have been well-studied in *Arabidopsis* (Leivar and Quail 2011) and characterized in the model plants tomato (*Solanum lycopersicum*), rice (*Oryza sativa*) and maize (*Zea mays* L.) (Nakamura et al. 2007; Kumar et al. 2016; Rosado et al. 2016). No petunia PIFs have been identified. In *Arabidopsis*, the PIF family has seven members: PIF1 and PIF3–8 (Leivar and Quail 2011). Members of the best-studied quartet—PIF1, PIF3, PIF4 and PIF5—act redundantly in the regulation of numerous physiological processes, among them, hypocotyl growth, leaf senescence and circadian rhythms (Leivar et al. 2008; Shor et al. 2017). PIFs not only transmit light cues, they also act as integrators of environmental and endogenous (phytohormone signaling, plant metabolic status) signals (Paik et al. 2017; Pham et al. 2018; Shor et al. 2018). PIFs intersect with the auxin, ethylene, gibberellin (GA), brassinosteroid, and jasmonic acid pathways to coordinate plant growth and development (De Lucas and Prat 2014; Leivar and Monte 2014). PIF4 (and to a lesser extent, PIF5) has been identified as a convergence point for the transduction of various signals (Quint et al. 2016; Zhao and Bao 2021). Moreover, PIF4 binds to a much higher number of DNA targets than PIF3 or PIF5 (Zhang et al. 2013), supporting the implication of PIF4’s control of a broader spectrum of biological processes. PIF functioning is mediated by DELLAs—repressors of GA signaling. DELLA-induced protein degradation has been shown for the PIF quartet members (De Lucas et al. 2008; Feng et al. 2008; Li et al. 2016). DELLAs also physically interact with PIF4 and PIF3, preventing their binding to target promoters (Feng et al. 2008). Interestingly, petunia DELLAs (PhDELLAs) are involved in the regulation of floral volatile production—they activate the expression of scent-related genes, whereas GA, known to promote the degradation of DELLA proteins (Sun 2010), suppresses scent (Ravid et al. 2017).

In view of the involvement of PIF family members in the coordination of numerous signals, including those shown to impact petunia scent (GA, light, temperature) (Cna’ani et al. 2015a; Ravid et al. 2017; Shor et al. 2023a), their involvement—and specifically that of PIF4 homologues—in the regulation of volatile production seems plausible. Here we identified petunia PIF family members and revealed that PhPIF4/5 positively regulates floral scent production by activating the expression of VOC-biosynthesis genes. The mechanism of this regulation includes promoter activation of the dawn-expressed positive regulator of scent, *EOBII*, suggesting PhPIF4/5’s involvement in initiating the scent-production regulatory system at daybreak; and upregulation of *PhDELLA*s, which suggests participation of PhPIF4/5 in GA-signal modulation of the VOC-biosynthesis machinery.

## Materials and methods

### Plant material and growth conditions

*Petunia × hybrida* line Mitchell diploid (W115) plants were grown in a glasshouse under 25 °C day/20°C night temperatures with a 16 h light/8 h dark photoperiod. For analysis of *PhPIF4/5* expression under far-red light and in the dark, flowers were detached from the plants and placed in a growth chamber (Percival) at 22 °C, under far-red/dark (FR, FR: 730 ± 10 nm, 20 µmol m^− 2^ s^− 2^) lighting conditions with 16 h light/8 h dark photoperiod, or in constant darkness (D). Light was provided by light-emitting diodes (LED30-HL1). The *Arabidopsis thaliana* (ecotype Columbia[Col]-0) PIF-overexpressing (PIF-OX) transgenic lines and *pifQ* (*pif1pif3pif4pif5*) mutants were described previously (Shor et al. 2017, 2018). *Arabidopsis* seeds were imbibed and cold-treated at 4 °C for 4 days, then sown on Petri dishes with Murashige and Skoog (MS) medium (Duchefa Biochemie, Netherlands) with 2% (w/v) sucrose. Plants were entrained in 14 h light:10 h dark with 100 µmol m^− 2^ s^− 1^ white light (supplied by Philips fluorescent lights TLD 18 W/840) at 23 °C for 8 or 10 days before being transferred for 2 days to continuous light (LL) or DD, respectively. The 10-day-old (for LL conditions) or 12-day-old (for DD conditions) seedlings were sampled for RNA extraction.

### Transient suppression of *PhPIF4/5 using TRV vectors*

Localized transient suppression of *PhPIF4/5* was performed using tobacco rattle virus (TRV) as described previously (Shor et al. 2023a). To generate pTRV2-PhPIF4/5, 145 bp of *PhPIF4/5* was amplified from cDNA using primers 5’-AGGAGCCGTGCTGCAGAA-3’, 5’-CATCTAGCATTGATGCTTTATC-3’ and inserted into pTRV2. As a control, pTRV2 carrying a fragment of *CHALCONE SYNTHASE* (pTRV2-CHS), shown previously not to affect floral VOC production, was used (Spitzer et al. 2007). pTRV2‐PhPIF4/5 or pTRV2‐CHS was introduced into *Agrobacterium tumefaciens* strain AGLO and mixed with agrobacteria carrying pTRV1 in a 1:1 ratio (in inoculation solution containing 200 µM acetosyringone and 10 mM MgCl_2_) prior to inoculation of flower petals at anthesis. Agroinfiltrated petal regions of 2 day postanthesis (dpa) or 3 dpa flowers were used for the experiments.

### Transient overexpression of ***PhPIF4/5***

For overexpression of *PhPIF4/5*, the CDS was amplified from petunia cDNA using primers 5’-ATGAACCCTTGTCTTCCTGAA-3’, 5’-CTAAAAATGTTTATGGGCT-3’ and cloned into a binary vector under a 35 S promoter. *Agrobacterium tumefaciens* strain AGLO was transformed with pCGN1559-35Spro:*PhPIF4/5* (PhPIF4/5-OX) or pDGB3α2-35Spro:*DsRED* (DsRED-OX) used as a control, and then injected into petals at anthesis. Agroinfiltrated petal regions of 2 dpa flowers were used for the experiments.

### Collection of emitted VOCs

Emitted floral scent compounds were collected for 24 h by localized headspace from the agroinfiltrated petal regions of flowers at 2 dpa (Skaliter et al. 2021). Glass tubes containing 100 mg Porapak Type Q polymer held in place with a plug of silanized glass wool were used as columns. Volatiles were eluted by 1.35 mL hexane + 0.45 mL acetone, and 2 µg isobutylbenzene was added to each sample as an internal standard, followed by GC-MS (Shor et al. 2023a). The experiments were performed in 3–4 biological repeats with similar results.

### Gene expression analysis

RNA was extracted from agroinfiltrated petal regions using the Tri-Reagent kit (Sigma‐Aldrich) and treated with RNase‐free DNase I (Thermo Fisher Scientific) prior to cDNA synthesis using ImProm‐II (Promega) reverse transcriptase and oligo(dT) primers. Two-step real‐time quantitative PCR (qRT-PCR) was performed on a CFX Opus 384 Real-Time PCR System (Bio-Rad) using 2X qPCRBIO SyGreen Blue Mix Hi-ROX (PCR Biosystems). Raw transcript level data were normalized to *EF1α*. For *Arabidopsis* samples, *tubulin* was used as the reference gene. Quantification calculations were carried out using the 2^−ΔΔCT^ formula as described (Nozue et al. 2007). The primers are shown in Supplementary Table S1. The experiments were performed in 2–3 biological repeats with similar results.

### Promoter activation assay

To test for activation of the *EOBII* promoter by PhPIF4/5, *DsRED* CDS was cloned under the 1345-bp promoter region of *EOBII* (*EOBII*pro) or under a mutated *EOBII* promoter lacking the G-box motif *CACGTG* (*EOBIIm*pro) into pCGN1559-*35S*pro:*PhPIF4/5* or pCGN1559 without *PhPIF4/5* (control). *Agrobacterium tumefaciens* strain AGLO was transformed with each of the obtained plasmids: pCGN1559-*35S*pro:*PhPIF4/5-EOBII*pro:*DsRED*, pCGN1559-*35S*pro:*PhPIF4/5-EOBIIm*pro:*DsRED*, pCGN1559-*EOBII*pro:*DsRED.* Each of these bacteria was co-infiltrated into petals of petunia flowers at anthesis or into leaves together with agrobacteria carrying pART27-*35S*pro:*YFP*. Inoculated tissues were analyzed ca.72 h after agroinfiltration. Transcriptional activation of *EOBII* promoter was evaluated in inoculated petal regions by DsRED fluorescence level or by *DsRED* mRNA level. YFP was used as a normalization factor. For imaging of DsRED and YFP fluorescence, a fluorescence binocular microscope (FLOUIII; Leica) was used, and UV light with DsRED and YFP filters was applied. The levels of YFP and DsRED signal were measured by ImageJ software (Mean Gray Value). mRNA levels of *DsRED* and *YFP* were analyzed by qRT-PCR. The experiments were performed three times with similar results.

### Bioinformatics analyses and tools

A phylogenetic tree, based on multiple protein sequence alignment, was constructed using MEGA11 (https://www.megasoftware.net/) (Tamura et al. 2021). The accession numbers of *Arabidopsis thaliana* and *Solanum lycopersicum* PIF proteins were obtained from (Leivar and Quail 2011; Rosado et al. 2016), respectively, and the sequences were downloaded from TAIR (https://www.arabidopsis.org/) and Sol Genomics Network (https://solgenomics.net) databases. Pairwise protein sequence alignment was conducted with the Needleman–Wunsch algorithm using the EMBOSS Needle package (https://www.ebi.ac.uk/Tools/psa/emboss_needle) by BLOSUM62 matrix.

## Results

### Petunia PIFs

**Fig. 1:**
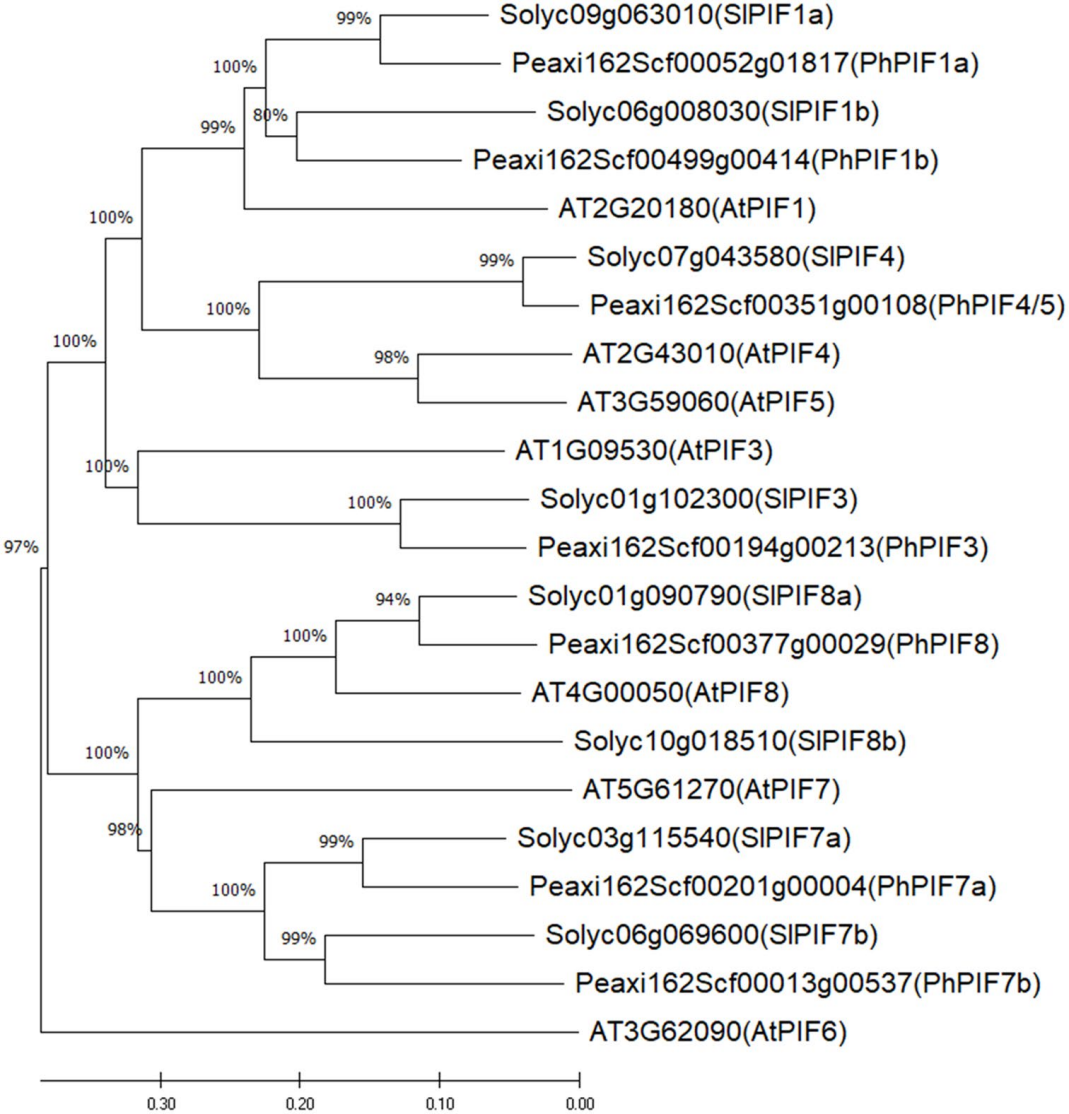
Phylogeny of petunia PIFs (PhPIFs). Neighbor-joining tree based on degree of sequence similarity between Petunia Axillaris (Peaxi), Solanum lycopersicum (Slyc) and Arabidopsis thaliana (AT) PIF proteins, built using the p-distance method with partial deletion option applied. Scale length represents number of substitutions per site. Numbers on the branches indicate percentage of replicate trees in which the associated taxa clustered together in the bootstrap test (1000 replicates)

PIFs of *S. lycopersicum* (Rosado et al. 2016) which, like petunia, belongs to the Solanaceae family, were used to identify putative petunia PIFs. Using TBLASTN against the CDSs predicted from the *Petunia axillaris* genome (https://solgenomics.net), the top hits were found and presence of the bHLH domain in these proteins was verified by PROSITE tool (https://prosite.expasy.org). This search resulted in seven putative petunia PIFs (PhPIFs). Neighbor-joining phylogenetic tree, based on multiple protein sequence alignment by ClustalW, was generated for the PhPIFs and those previously characterized in model plants *A. thaliana* and *S. lycopersicum* (Fig. 1). In petunia, similar to PIFs in tomato, the homologues of the *Arabidopsis* central PIF quartet (homology with AtPIFs was confirmed by reciprocal BLASTp) were: PhPIF1a and PhPIF1b, which were most closely related to AtPIF1; PhPIF3 which was homologous to AtPIF3; and the protein termed PhPIF4/5 that clustered with AtPIF4 and AtPIF5. Pairwise protein sequence alignment between PhPIF4/5 and AtPIF4 or AtPIF5 revealed similar sequence identity (32%), supporting PhPIF4/5’s relation to both of these *Arabidopsis* PIFs.

Next, we evaluated accumulation of PIF transcripts in petunia petals using previously generated RNA-seq data (Shor et al. 2023b). *PhPIF1b* and *PhPIF7b* were not expressed in petals of mature flowers, but their transcripts could be found in the leaf transcriptome (Villarino et al. 2014) available at https://solgenomics.net/, as transcripts comp20642 and comp33265, respectively. All other petunia PIFs—*PhPIF1a*, *PhPIF3*, *PhPIF4/*5, PhPIF7a and *PhPIF8*—were expressed in the corolla. Characterization of temporal changes in PIF mRNA levels, based on RNA-seq data, revealed that in 1 dpa flowers, *PhPIF3* and *PhPIF7a* transcripts are higher accumulated in the morning, *PhPIF8* is higher in the evening. Expression levels of *PhPIF1a* and *PhPIF4/5* did not differ at these time points (Supplementary Fig. S1).

**Fig. 2:**
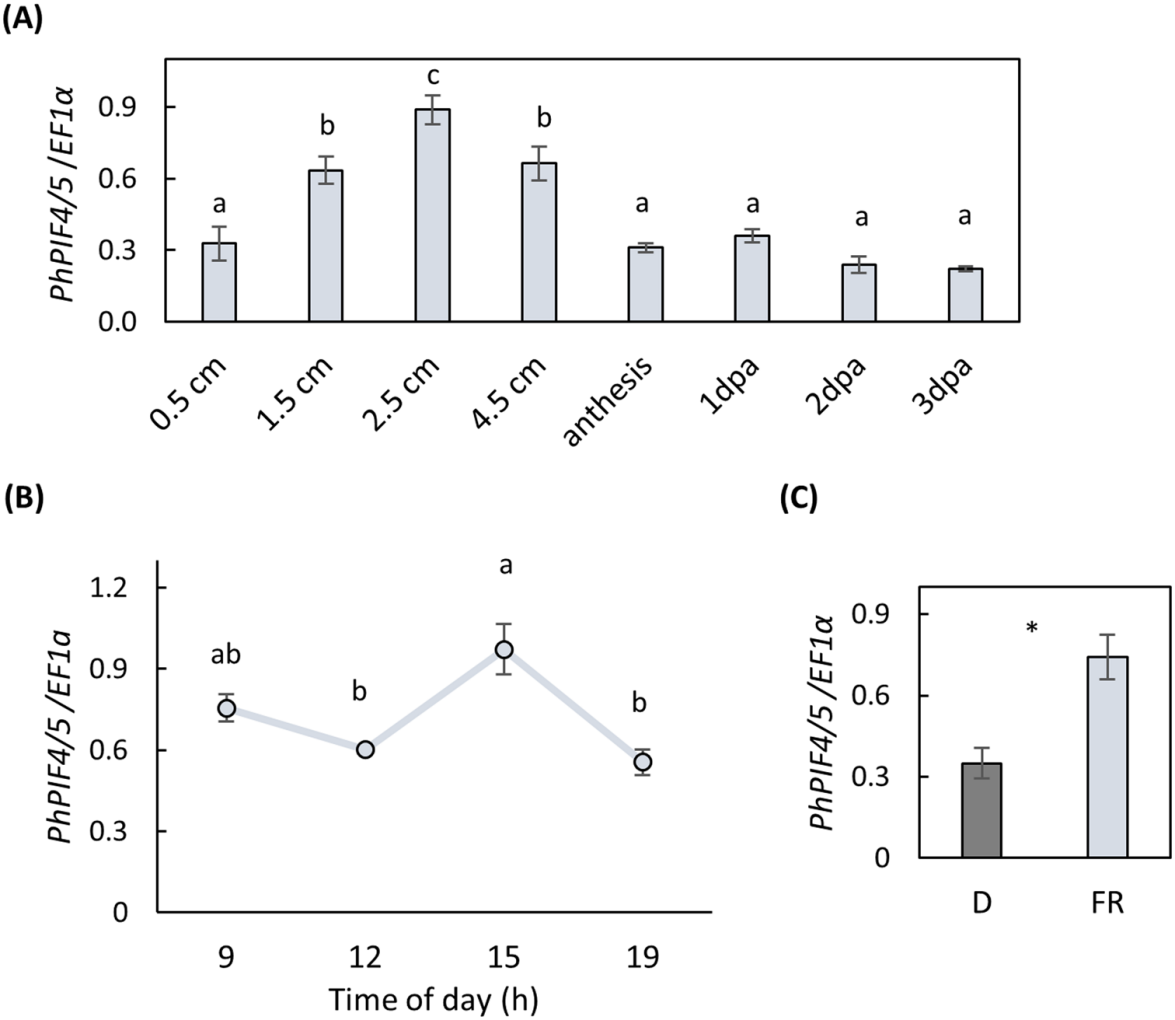
Transcript levels of PhPIF4/5 in petunia petals. (**A**) Levels of PhPIF4/5 (at 15.00 h) in developing flowers, length of buds is indicated. (**B**) PhPIF4/5 expression during daytime in flowers at anthesis. Plants were grown under 16-h light/8-h dark photoperiod (lights on 06.00 h/lights off 22.00 h). For statistical analysis in (A) and (B), one-way ANOVA with post-hoc Tukey HSD test was applied (*P* ≤ 0.05). (**C**) Effect of far-red light on PhPIF4/5 expression. Flowers at anthesis were exposed to far-red/dark conditions (16-h light/8-h dark photoperiod) or to constant dark for 2 days. Samples were collected from flowers in darkness (**D**) or under far-red light (FR) at 11.00 h. Significance of differences between treatments was calculated using Student’s t-test, *P ≤ 0.05. EF1α was used as an internal reference gene. Data are means ± SEM, *n* = 3–4

According to the *Arabidopsis* model, PIF4 acts as a hub, integrating numerous environmental and developmental signals, including those regulating floral scent (Quint et al. 2016; Paik et al. 2017). Based on this we reasoned that PhPIF4/5 may also play a role in regulation of VOC production. Focusing on PhPIF4/5 for the further analysis, we performed detailed developmental and diurnal expression profiling of PhPIF4/5 by qRT-PCR. This analysis revealed an increase in transcript level with bud development to a peak in 2.5 cm buds, then a gradual decrease (Fig. 2A). During the daytime, *PhPIF4/5* mRNA levels peaked in the afternoon in mature flowers (Fig. 2B), similar to the orthologs in tomato (SlPIF4) and *Arabidopsis* (PIF4) which are highly expressed in the middle of the day with a peak at around ZT8 (Soy et al. 2014; Rosado et al. 2016).

In *Arabidopsis*, expression of *PIF4* and *PIF5* is induced by red and far-red light (Huq and Quail 2002; Oh et al. 2020). To test whether the biological properties of *PhPIF4/5* are similar to those of the *Arabidopsis* homologues, we examined *PhPIF4/5* expression in response to far-red lighting. Petunia flowers at anthesis were placed under far-red/dark (FR) conditions or in constant darkness (D), and mRNA levels of *PhPIF4/5* were evaluated 2 days later. Expression of *PhPIF4/5* was significantly higher under FR vs. D conditions (Fig. 2C), demonstrating a far-red-sensitive expression pattern analogous to that in *Arabidopsis*.

### PhPIF4/5 positively affects petunia floral scent production

**Fig. 3:**
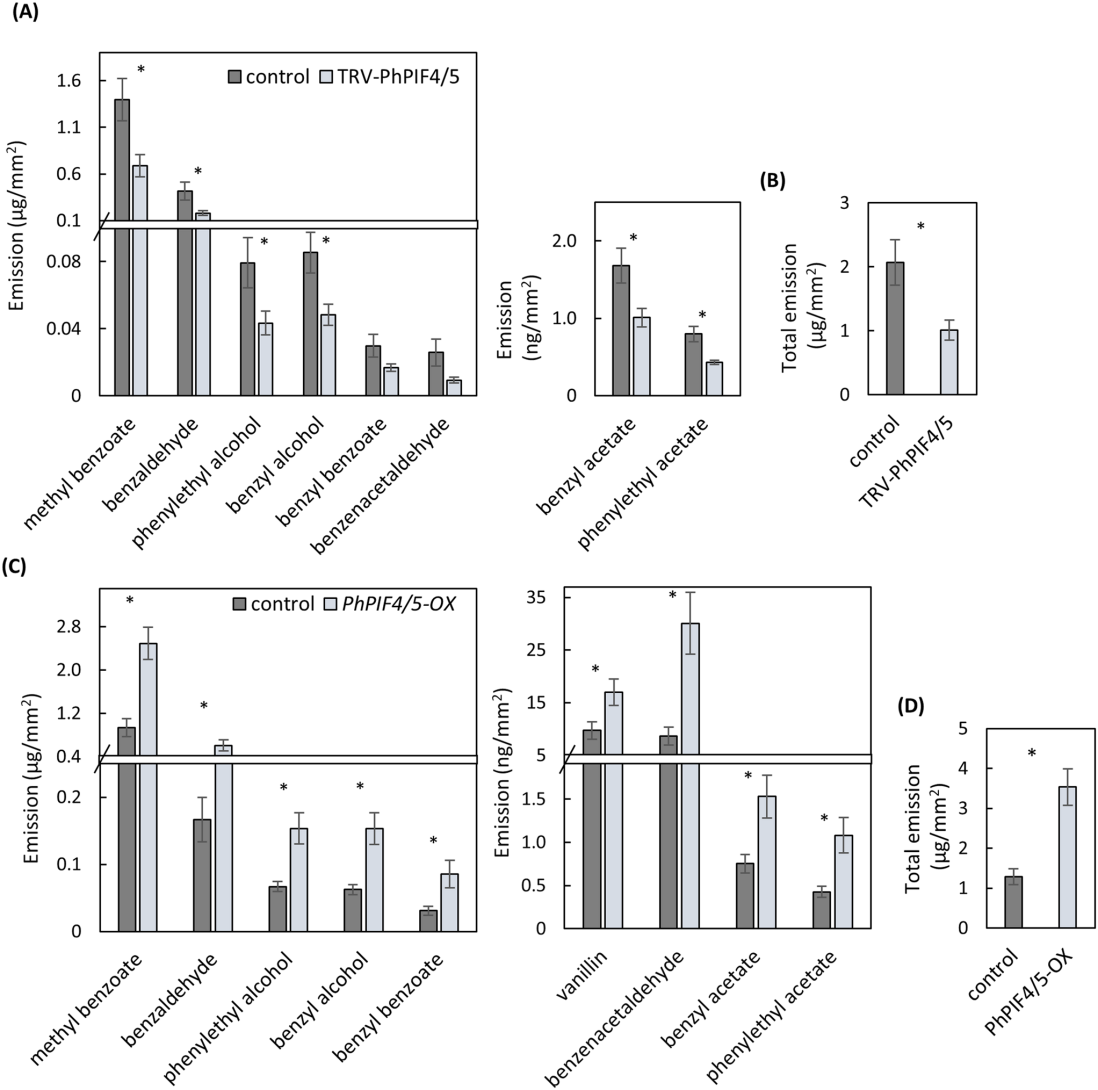
*PhPIF4/5* activates petunia floral scent production. Petals were inoculated with *Agrobacterium* carrying (**A, B**) TRV-PhPIF4/5 for transient suppression of *PhPIF4/5* or with TRV-CHS as a control, or (**B, C**) binary vector with 35 S:*PhPIF4/5* (*PhPIF4/5 -OX*) for transient overexpression of *PhPIF4/5* or with 35 S:*DsRED* as a control. Emission levels of volatiles: (**A, C**) individual scent compounds, (**B, D**) total emission, *n* = 5–7. Data are means ± SEM. Significance of differences between treatments was calculated using Student’s t-test, *P ≤ 0.05

To examine the involvement of PhPIF4/5 in the regulation of floral scent production, *PhPIF4/5* was locally and transiently suppressed in petunia petals by virus-induced gene silencing (VIGS) using TRV (Shor et al. 2023a). pTRV2 plasmids with a fragment of *PhPIF4/5* (TRV-PhPIF4/5) or without it (control) were agroinfiltrated into the petals. A ca. 5-fold reduction in *PhPIF4/5* mRNA accumulation in the inoculated areas was confirmed by qRT-PCR (Supplementary Fig. S2A). Two days post-infiltration, the levels of emitted VOCs were evaluated by localized headspace followed by GC-MS. The levels of the most abundant scent compounds—methyl benzoate, benzaldehyde, phenylethyl alcohol, benzyl alcohol—decreased in response to *PhPIF4/5* suppression (Fig. 3A). Consequently, total VOC emission from infected areas was 2 times lower in TRV-PhPIF4/5 petals than in controls (Fig. 3B).

To further confirm the involvement of PhPIF4/5 in scent regulation, *PhPIF4/5* was locally and transiently overexpressed in petals by inoculation with agrobacteria carrying *PhPIF4/5* under the *35 S* promoter (*PhPIF4/5-OX)* or *DsRED* under *35 S* promoter (*DsRED-OX*), used as a control. Agroinfiltration with *PhPIF4/5-OX* led to a three orders of magnitude increase in *PhPIF4/5* transcript levels compared to the control (Supplementary Fig. S2B). Localized headspace analysis of the infiltrated regions revealed that emission of all of the main scent compounds was significantly higher in *PhPIF4/5* -overexpressing tissues than in controls. Total emission from *PhPIF4/5-OX* petals was ca. 3.5 times higher than from peals, inoculated with *DsRED-OX* (Fig. 3C, D). Taken together, these results indicated that PhPIF4/5 positively regulates floral VOC emission.

### PhPIF4/5 activates expression of scent-related genes

**Fig. 4:**
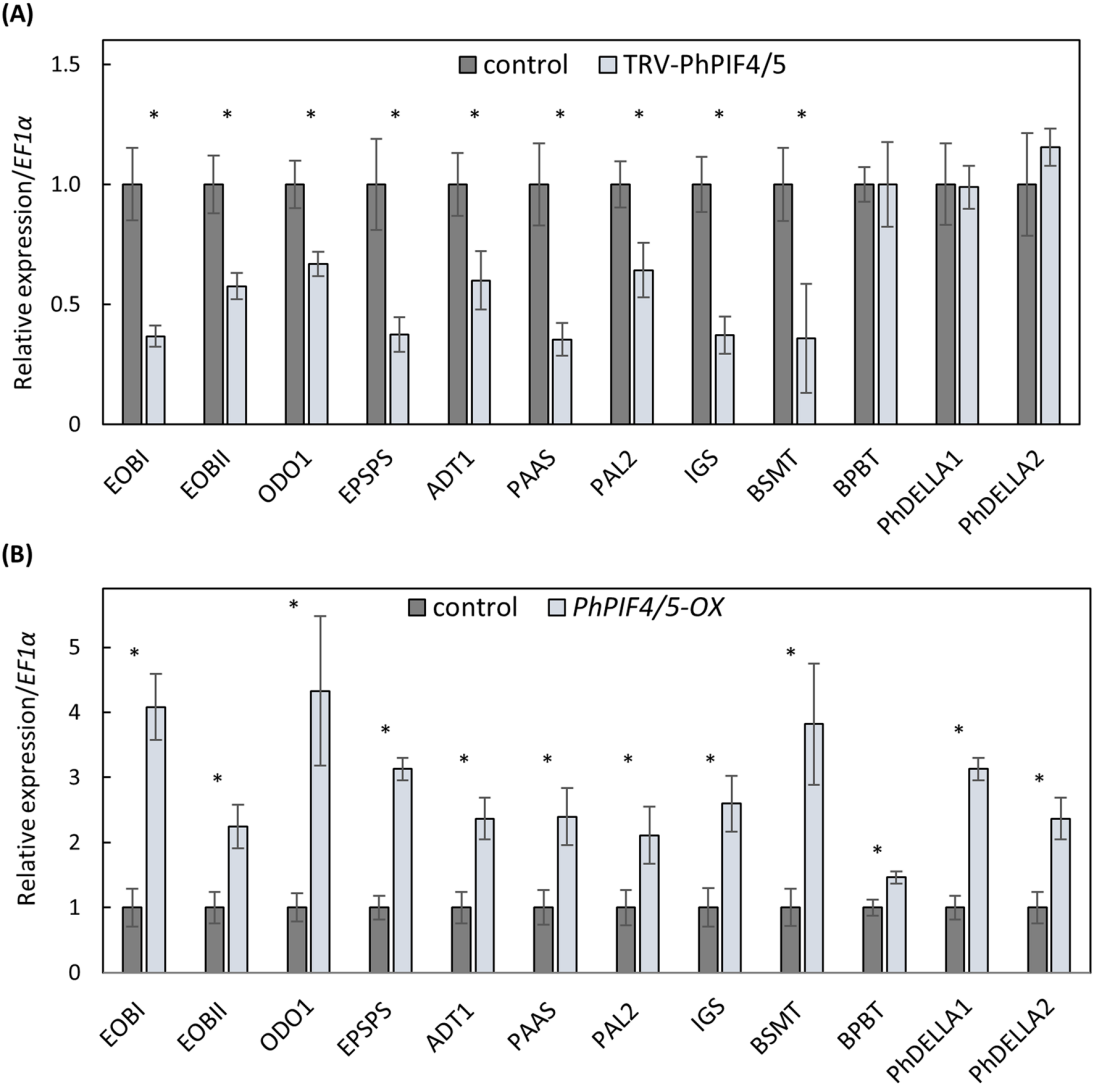
*PhPIF4/5* positively affects expression of scent-related genes. Transcript levels were evaluated in petal tissues agroinfiltrated with (**A**) TRV-PhPIF4/5 for transient suppression of *PhPIF4/5* or with TRV-CHS as a control, (**B**) 35 S:*PhPIF4/5* (*PhPIF4/5 -OX*) for transient overexpression of *PhPIF4/5* or with 35S:*DsRED* as a control. Samples were collected at 11.00 h, *n* = 5–6. *EF1α* was used as an internal reference gene. Expression levels of each target was normalized to that in the control. Data are means ± SEM. The significance of differences between treatments was calculated using Student’s t-test: *P ≤ 0.05

To deeply characterize the positive effect of PhPIF4/5 on scent production, transcript levels of the known regulators of scent and of the genes involved in VOC biosynthesis were analyzed in inoculated petal areas. The samples for analysis were collected in the morning as PIF4 and PIF5 often act as dawn-active inducers of gene expression (Seaton et al. 2018). A qRT-PCR analysis revealed that TRV-based *PhPIF4/5* silencing causes a significant reduction in mRNA levels of scent-related genes (Fig. 4A). The genes encoding biosynthesis enzymes of the phenylpropanoid pathway (*PAAS*, *PAL2*, *BSMT*, *IGS*), shikimate pathway (*EPSPS*, *ADT1*), and activators of VOC biosynthesis (*EOBI*, *EOBII*, *ODO1*) showed lower expression in TRV-PhPIF4/5 than in the TRV control. Unlike all other tested VOC-biosynthesis genes, *BPBT* was not affected in TRV-PhPIF4/5-inoculated petals. Suppression of *PhPIF4/5* also did not affect positive regulators of scent *PhDELLA*s.

In line with *PhPIF4/5* silencing, transient overexpression of *PhPIF4/5* caused a 2- to 4-fold increase in transcript accumulation of regulators *EOBI*, *EOBII* and *ODO1*. The mRNA levels of all tested biosynthesis genes were also significantly higher in *PhPIF4/5-OX* petals than in *DsRED-OX* controls (Fig. 4B). Transcript levels of both *PhDELLA1* and *PhDELLA2* were significantly increased in response to *PhPIF4/5* overexpression, indicating the involvement of PhDELLAs in PhPIF4/5-mediated activation of VOC production. These data suggest that PhPIF4/5 enhances scent production via activation of the expression of positive regulators of scent production and VOC-biosynthesis genes.

**Fig. 5:**
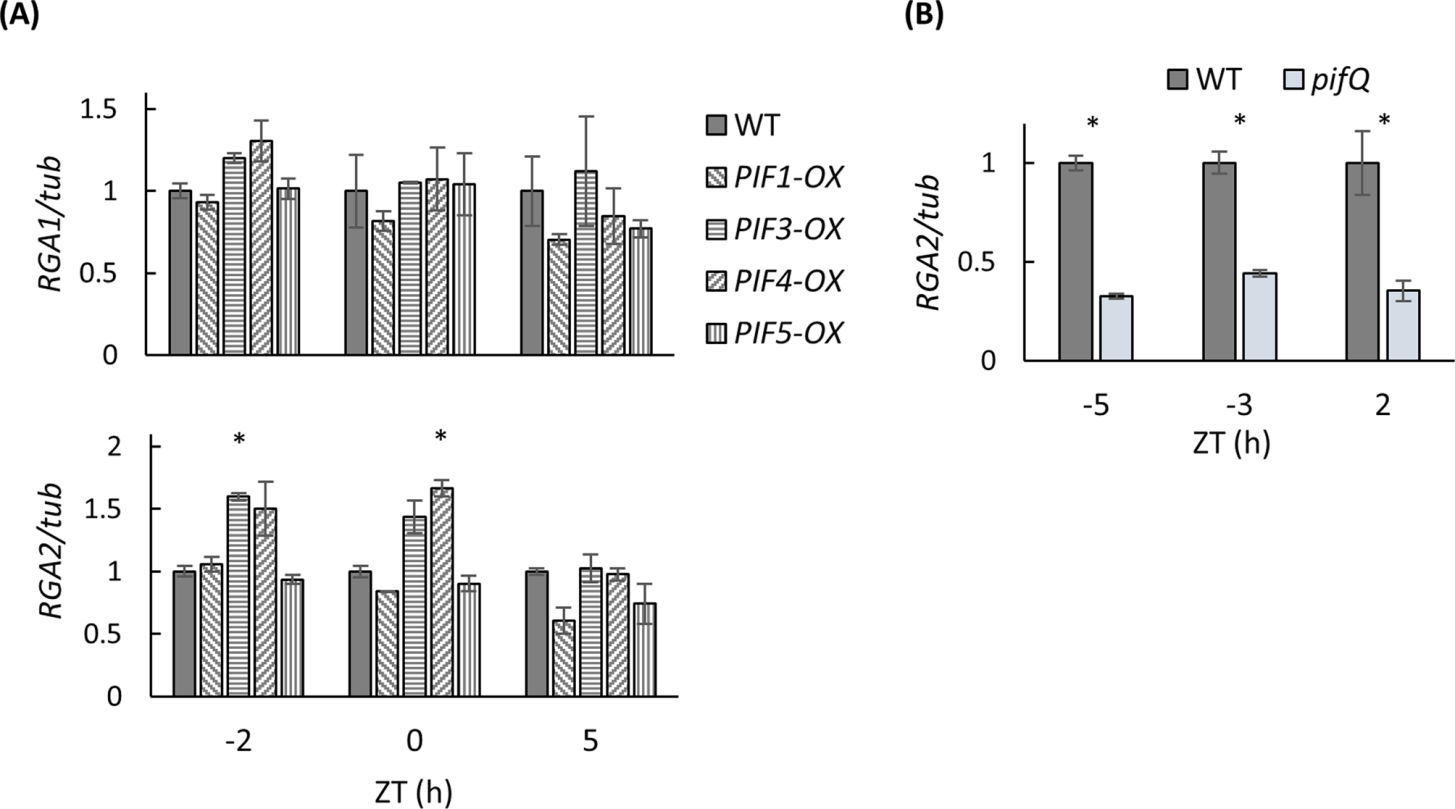
PIF4 activates expression of *DELLA* in *Arabidopsis* seedlings. Plants were entrained in 14 h light/10 h dark before being transferred to free running conditions. (**A**) Independent *PIF-*overexpressing (*PIF-OX*) transgenic and wild-type (WT) control lines were sampled in constant light (LL). (**B**) *pifQ (pif1pif3pif4pif5)* mutant and WT control plants sampled in constant darkness (DD). Samples were collected at the indicated times for qRT-PCR. *n* = 3. *Tubulin* (*tub*) was used as an internal reference gene. Expression of the targets was normalized to that in the WT at each time point (ZT). Data are means ± SEM. Significance of differences between treatments was calculated using Student’s t-test, *P ≤ 0.05

To further confirm the previously unreported effect of PIFs on DELLA expression, and to test the generality of the PIFs’ influence on *DELLA*s, we used *Arabidopsis* (Col-0) transgenic plants overexpressing PIF quartet members *PIF1*, *PIF3*, *PIF4* or *PIF5* (Shor et al. 2018). Transcript levels of *REPRESSOR OF GA 1* (*RGA1*) and *RGA2*, homologous to *PhDELLA1* and *PhDELLA2* (Shor et al. 2023a), in PIF-overexpressing seedlings were evaluated under constant light conditions (LL) at different times of the day—before subjective dawn (ZT-2), at dawn (ZT0) and in the middle of the day (ZT5) (Fig. 5A). *RGA1* was not affected by overexpression of any of the PIF quartet members. In contrast, *RGA2* expression was responsive to *PIF3* and *PIF4*: it increased at dawn and before dawn in *PIF4-* and *PIF3-*overexpressing seedlings, respectively, as compared to wild-type (WT) plants. Due to the functional redundancy among PIFs, the effect of individual PIFs’ suppression on DELLA expression was not evaluated. Instead, the effect of PIFs on *RGA2* expression was additionaly confirmed in a quadruple *pif* mutant (*pifQ*) at different time points in constant darkness (DD) (Fig. 5B). *RGA2* mRNA levels were lower in *pifQ* than in the WT during the subjective night (ZT-5, ZT-3) and at the beginning of the subjective day (ZT2). These results suggest that PIFs positively affect *RGA2* expression independently of light/dark conditions, indicating that the PIFs’ activity in this regulation is probably not related to their role in light-signal transduction. Activation of DELLAs by PIF quartet members in different plants and under different lighting conditions (LL and DD) further supports the impact of PIFs in DELLA-regulated scent-related processes.

### PhPIF4/5 transcriptionally activates EOBII

**Fig. 6:**
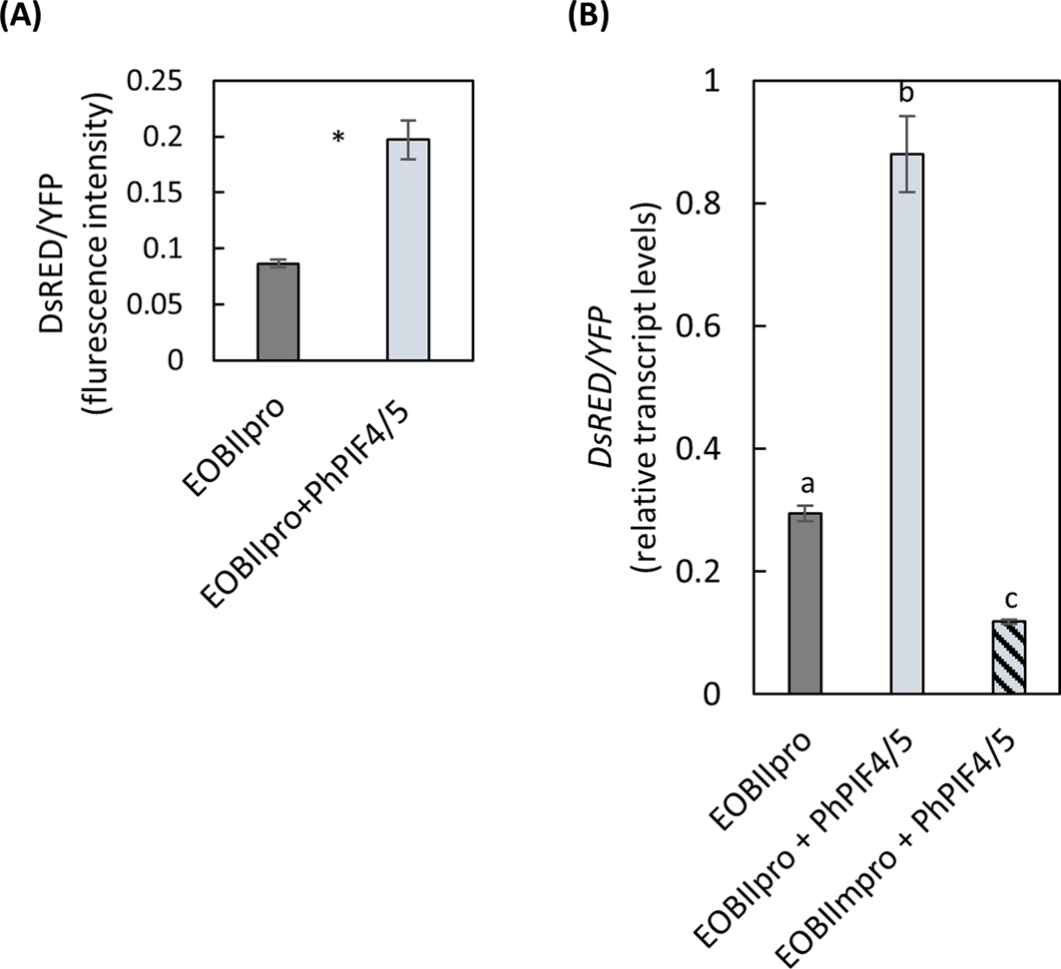
*EOBII* promoter is activated by PhPIF4/5 in petunia flowers. Petals were agroinfiltrated with binary vector carrying *DsRED* under native (*EOBII*pro) or mutated (lacking G-box; *EOBIIm*pro) *EOBII* promoter and *35 S*:*PhPIF4/5* (+ *PhPIF4/5*). As a control, binary vector without PhPIF4/5 (*EOBII*pro) was used. For normalization, petals were co-infiltrated with a vector carrying *35 S*:*YFP*. (**A**) Relative DsRED levels, estimated by fluorescence in petal tissues expressing *DsRED* under the native *EOBII* promoter. Tissues were imaged by fluorescence microscope and the DsRED/YFP ratio was calculated (*n* = 6). Significance of differences between treatments was calculated using Student’s t-test, *P ≤ 0.05. (**B**) Effect of PhPIF4/5 on expression levels of *DsRED* measured by qRT-PCR in petal tissues expressing *DsRED* under the native or mutated *EOBII* promoter. Samples were collected at 12.00 h (*n* = 4). Expression of *DsRED* was normalized to *YFP* transcript levels and then to the maximum for all samples in the experiment. For statistical analysis, one-way ANOVA with post-hoc Tukey HSD test was applied (*P* ≤ 0.05). Data are means ± SEM.

*Arabidopsis* PIFs 4 and 5, homologues of PhPIF4/5, are accumulated at the end of night (Leivar and Monte 2014) and implement their regulatory effect on their target genes’ expression at around dawn (Seaton et al. 2018). Among the known regulators of petunia floral VOC production, *EOBII* and *EOBI* are expressed at dawn/in the early morning (Spitzer-Rimon et al. 2010, 2012). To evaluate whether morning activation of *EOBI* and/or *EOBII* expression is related to PhPIF4/5 activity, we examined the presence of PIF-binding motifs—G-box (CACGTG) and/or PBE-box (CACATG) (Hornitschek et al. 2012; Zhang et al. 2013)—in the promoter sequences (2000 bp upstream of ATG) of *EOBI* and *EOBII*. *EOBI* promoter did not contain any of these motifs, whereas *EOBII* promoter contained G-box at -850 bp. To examine the ability of PhPIF4/5 to activate the *EOBII* promoter, we agroinfiltrated petals with the coding sequence of *DsRED* fluorescent protein under the *EOBII* promoter (*EOBII*pro:*DsRED*) together with *35S*pro:*PhPIF4/5* (*EOBII*pro + PhPIF4/5) or without it (*EOBII*pro, control), and with 35Spro:*YFP* used for normalization. Activity of the *EOBII* promoter was evaluated by relative DsRED/YFP fluorescence signal, detected by image analysis. Transient localized overexpression of *PhPIF4/5* (*EOBII*pro + PhPIF4/5) caused a 2-fold increase in the DsRED/YFP ratio, compared to the control (*EOBII*pro) (Fig. 6A), indicating activation of the *EOBII* promoter by PhPIF4/5. The ratio between DsRED and YFP was evaluated at the transcript level by qRT-PCR as well, considering that this method provides more accurate results, as DsRED accumulation in cells can be reflected by different dynamics in promoter activity and protein degradation. These experiments also revealed a strong increase in DsRED mRNA level as a result of *35S*pro:*PhPIF4/5* expression (Fig. 6B). Moreover, *EOBII* promoter was activated by transient overexpression of *PhPIF4/5* in petunia leaves as well (Supplementary Fig. S3A, B). To validate the importance of G-box in PIF4/5-mediated activation of EOBII, we generated a mutated *EOBII* promoter that lacks this motif (*EOBIIm*pro). Overexpression of *PhPIF4/5* in petals together with *DsRED* expressed under the mutated *EOBII* promoter (*EOBIIm*pro + PhPIF4/5) or under the native *EOBII* promoter (*EOBII*pro + PhPIF4/5) revealed that this mutation abolishes the promoter’s activation by PhPIF4/5 (Fig. 6B). These data suggest that PhPIF4/5 activates the *EOBII* promoter and that the presence of the G-box motif affects this activation.

## Discussion

The scent-production machinery in petunia is sensitive to light, temperature, the circadian clock and the phytohormones GA and ethylene (Underwood et al. 2005; Cna’ani et al. 2015a; Fenske et al. 2015; Ravid et al. 2017; Shor et al. 2023a). PIFs are known to be involved in the transduction and integration of these environmental and developmental signals (Leivar and Monte 2014; Paik et al. 2017). Interestingly, anthocyanin pigmentation, which originates from the same phenylpropanoid pathway as petunia floral VOCs and is shown to be coregulated with them (Zvi et al. 2008; Cna’ani et al. 2015b), is sensitive to PIFs (Liu et al. 2015; Seaton et al. 2018). Numerous processes are redundantly regulated by PIFs; e.g., overlapping functions of PIF quartet members have been shown for photomorphogenesis, the transition to scotomorphogenesis and circadian clock entrainment (Shin et al. 2009; Shor et al. 2017). PIF1 and PIF3 negatively regulate chlorophyll biosynthesis and chloroplast development (Shin et al. 2009; Stephenson et al. 2009). PIF3 and PIF4 integrate GA and light signals to modulate hypocotyl growth (De Lucas et al. 2008; Feng et al. 2008). The latter has been specifically shown to incorporate additional signaling pathways (Choi and Oh 2016). For example, in the control of hypocotyl elongation, PIF4 integrates sensitivity to light, temperature, brassinosteroid signals and, together with the circadian clock, provides time-gating of auxin biosynthesis for growth initiation (Koini et al. 2009; Oh et al. 2012; Zhu et al. 2016; Pereyra et al. 2022). Functions of PIF4 and PIF5 largely overlap, and they often work collaboratively in the same pathways, among them: transmission of output signals from the circadian oscillator, regulation of ethylene signaling and biosynthesis during leaf senescence, shade avoidance and anthocyanin biosynthesis (Nozue et al. 2007; Lorrain et al. 2008; Sakuraba et al. 2014; Liu et al. 2015; Liebsch and Keech 2016). Yet, the mechanisms of PIF4 and PIF5 regulation of the same process can differ. For example, in inhibiting light-induced anthocyanin production, PIF5 suppresses late anthocyanin-biosynthesis genes by direct interaction with their promoters, whereas PIF4 directly represses expression of the positive regulator of anthocyanin biosynthesis *PRODUCTION OF ANTHOCYANIN PIGMENT 1* (Liu et al. 2015, 2021). Moreover, PIF4 and PIF5 regulation of the phenylpropanoid pathway, i.e., anthocyanin production, in *Arabidopsis* seedlings is specific to growth conditions. Whereas under standard conditions, *pif4pif5* mutants display a photoperiod-dependent low-anthocyanin phenotype, indicating their positive effect on anthocyanin biosynthesis (Seaton et al. 2018), light-induced anthocyanin accumulation is inhibited by PIF4 and PIF5 (Liu et al. 2015, 2021). Although it has been extensively studied in vegetative organs, the role of PIF4 and PIF5 homologues in the regulation of phenylpropanoid biosynthesis in flowers has not been investigated.

The petunia PIF family is represented by seven members (PhPIFs) which are similar to those in tomato (Rosado et al. 2016) (Fig. 1). Five of them, including PhPIF4/5, are expressed in the corolla. The name PhPIF4/5 was given based on its similarity to the two *Arabidopsis* proteins, PIF4 and PIF5. The presence of one protein that is similar to both of these *Arabidopsis* PIFs has also been observed in other eudicots, and duplication in the PIF4 clade has been found specifically in several eudicots of the family Brassicaceae (Rosado et al. 2016).

Here we revealed the involvement of PhPIF4/5 in specialized metabolism during advanced stages of flower development, i.e., floral scent production. Both VOC-biosynthesis genes and positive regulators of the pathway were significantly activated by *PhPIF4/5*, leading to increased levels of the emitted volatiles. Moreover, PhPIF4/5 was shown to act upstream of the central activator of scent, *EOBII*. Whereas overexpression of *PhPIF4/5* increased transcript levels of all of the tested scent-related genes, suppression of *PhPIF4/5* did not affect *BPBT* or *PhDELLA*s (Fig. 4). This might be due to the redundancy among PIF family members, similar to that described for other PIF-regulated physiological processes (Leivar et al. 2012; Shor et al. 2017). The fact that PIF4, PIF5 and PIF3 often regulate the same target genes, as revealed by CHIP-seq (Oh et al. 2012; Zhang et al. 2013), highlights the overlapping functions of these PIFs and their tendency toward redundance. Interestingly, the pattern of *BPBT* expression also differed from that of other VOC-biosynthesis genes in experiments evaluating scent production in response to far-red light and to suppression of PHYA (Shor et al. 2023a). In those experiments, the genes encoding the main regulators of VOC biosynthesis, including *EOBII*, were upregulated in response to the tested conditions. However, *BPBT*, known to be activated by EOBII under normal conditions (Spitzer-Rimon et al. 2010), was not affected. This suggests the presence of an additional mechanism governing the control of *BPBT* expression, which is opposite to that mediated by EOBII.

PIFs 4 and 5 are dawn-active transcriptional factors (Seaton et al. 2018) as well as the major positive regulator of scent, *EOBII*. Activation of *EOBII* promoter, which contains a PIF-binding G-box motif, by PhPIF4/5 and the importance of G-box for this activation (Fig. 6) suggest that PhPIF4/5 is required for peak *EOBII* transcript accumulation at dawn. This subsequently initiates the cascade of the events—including activation of *ODO1* and expression of VOC-biosynthesis genes—resulting in the emission of volatiles in the evening. Similar PIF-mediated induction of a morning expression peak has been shown for the core circadian clock component *CIRCADIAN CLOCK ASSOCIATED 1* (*CCA1*) (Shor et al. 2017). Genes with a diurnal expression pattern similar to that of, e.g., *EOBII* and *CCA1*, are often controlled by both light-sensing regulators and a circadian oscillator (Seaton et al. 2018). Therefore, it can be suggested that *EOBII* is also under circadian control. Among the core components of the circadian oscillator, only the PSEUDO-RESPONSE REGULATOR (PRR) transcription factors bind specifically to G-box on the target promoters (Liu et al., 2016). Hence, it would be reasonable to evaluate the involvement of PRRs in the regulation of *EOBII* expression and floral scent production.

PhPIF4/5, similar to PhDELLAs—the transmitters of GA signaling—positively regulate scent and expression of VOC-biosynthesis and regulatory genes (Ravid et al. 2017). However, it is well established that at the protein level, DELLAs negatively affect PIF activity (De Lucas et al. 2008; Feng et al. 2008; Li et al. 2016). Here we provide evidence for the positive transcriptional/post-transcriptional effect of PIFs on DELLAs: of petunia PhPIF4/5 on the expression of *PhDELLA1* and *PhDELLA2* and of *Arabidopsis* PIF4 and PIF3 on the expression of *RGA2* (Figs. 4 and 5). Till now, transcriptional regulation of DELLAs by PIFs had only been demonstrated for *Arabidopsis* PIF1 (PIL5) which positively affects *RGA1* and *RGA2* expression in germinating seeds (Oh et al. 2007). The findings presented here expand our understanding of the mechanism of PIF4–DELLA fine-tuning and of PIF-mediated modulation of GA signaling (Supplementary Fig. S4), a model that includes the following feedback loops: PIF4, together with PIF5, is involved in the control of GA biosynthesis (Filo et al. 2015), and the latter negatively controls the abundance of DELLA proteins, leading them to degradation (Sun 2010); DELLAs induce protein degradation of PIFs (De Lucas et al. 2008; Feng et al. 2008; Li et al. 2016), while PIF4 activates *DELLA* expression. In the petunia corolla, the levels of GA—which acts as a repressor of floral scent production (Ravid et al. 2017)—decrease during flower development in parallel with increasing sensitivity to GA and initiation of scent emission (Patrick et al. 2021; Shor et al. 2023b). Considering the GA–DELLA–PIF4/5 interactions in petunia, demonstrated previously (Ravid et al. 2017) and in the current work, PhDELLA–PhPIF4/5 fine-tuning may be a part of the GA-sensitivity-adjustment mechanism in petals.

**Fig. 7:**
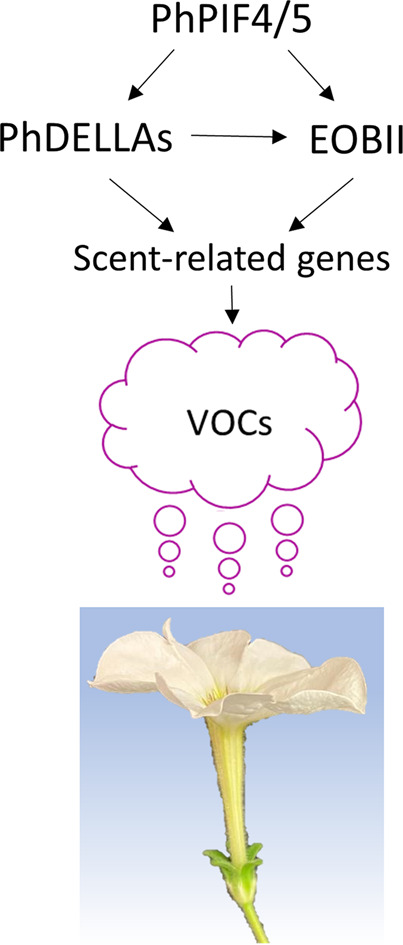
A model showing PhPIF4/5 input in petunia floral scent emission. Petunia PHYTOCHROME INTERACTING FACTOR 4/5 (PhPIF4/5) positively regulates VOC production and expression of scent-related genes. This includes transcriptional activation of the master regulator of scent *EMISSION OF BENZENOIDS II* (*EOBII*) and upregulation of *PhDELLA* transcripts

Taken together, we propose that the PhPIF4/5-mediated positive effect on VOC production includes transcriptional activation of both *EOBII* and *PhDELLA*s (Fig. 7). Even though PhDELLAs have been shown to increase *EOBII* expression (Ravid et al. 2017), PhPIF4/5’s effect on *EOBII* is not only exerted through PhDELLAs, because a reduction in scent was observed in flowers with suppressed *PhPIF4/5*, where *PhDELLA*s were not affected. Interestingly, VOC-biosynthesis genes can be activated by PhPIF4/5 in EOBII- and PhDELLAs- independent manner. For example, *PAAS* is not affected by EOBII (Spitzer-Rimon et al. 2010) and positively regulated by PhDELLAs (Ravid et al. 2017), however, *PAAS* level decreased in response to *PhPIF4/5* suppression, which did not cause reduction in *PhDELLA*s’ levels. Thus, PhPIF4/5 may link partially independent routes leading to scent production, allowing for precise regulation of scent-related genes. Considering the role of PhPIF4/5 in the regulation of scent, and the signaling-hub function of PIFs in the adjustment of numerous physiological processes (Leivar and Quail 2011; Choi and Oh 2016), an evaluation of PhPIF4/5’s participation in the integration of external and internal signals for the production of floral volatiles is warranted.

### Accession numbers

PhPIF1a (Peaxi162Scf00052g01817), PhPIF1b (Peaxi162Scf00499g00414), PhPIF3 (Peaxi162Scf00194g00213), PhPIF4/5 (Peaxi162Scf00351g00108), PhPIF7a (Peaxi162Scf00201g00004), PhPIF7b (Peaxi162Scf00013g00537), PhPIF8 (Peaxi162Scf00377g00029), EOBI (Peaxi162Scf00129g01231), EOBII (Peaxi162Scf00080g00064), PhDELLA1 (Peaxi162Scf00305g00129), PhDELLA2 (Peaxi162Scf00159g00167), RGA1 (AT2G01570), RGA2, GAI (AT1G14920).

### Electronic supplementary material

Below is the link to the electronic supplementary material.

Supplementary Material 1

## Data Availability

All new created data is already contained within this article and the supplementary materials.
