# Supplementary material for: Petunia PHYTOCHROME INTERACTING FACTOR 4/5 transcriptionally activates key regulators of floral scent
Source: Plant Mol Biol. 2024 May 30;114(3):66. doi: 10.1007/s11103-024-01455-8 (PMC11139750; doi:10.1007/s11103-024-01455-8)
Supplement: Supplementary file 1 — Supplementary Material 1 [file 11103_2024_1455_MOESM1_ESM.pdf]

Petunia PHYTOCHROME INTERACTING FACTOR 4/5 transcriptionally activates key regulators of floral scent.

Ekaterina Shor[1] and Alexander Vainstein[2]

[1] Institute of Plant Sciences, ARO, Volcani Institute, Rishon LeZion, Israel.

[2] The Robert H. Smith Institute of Plant Sciences and Genetics in Agriculture, The Hebrew University, Rehovot, Israel.

Corresponding author: Alexander Vainstein, ORCID ID 0000-0002-2667-3081

The Robert H. Smith Institute of Plant Sciences and Genetics in Agriculture, The Hebrew University, Rehovot, Israel.

Email: alexander.vainstein@mail.huji.ac.il

List of supplementary information Figures and Tables

Fig. S1. Expression profiles of petunia PIFs (PhPIFs).

Fig. S2. Relative expression levels of PhPIF4/5 in agroinfiltrated petunia petal regions.

Fig. S3. *EOBII* promoter is activated by PhPIF4/5 in petunia leaves.

Fig. S4. A model showing PIF4–DELLA fine-tuning in the modulation of GA signaling in *Arabidopsis*.

Table S1. Primers used for qRT-PCR analyses.

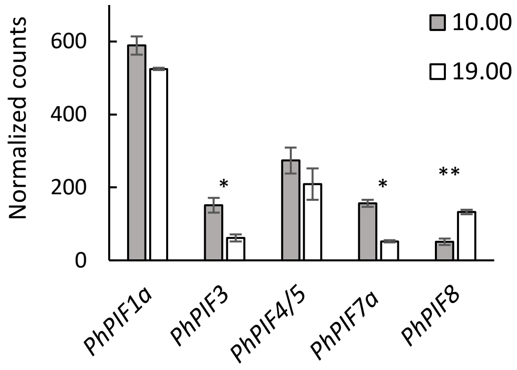

Supplementry Figure 1. Expression profiles of petunia PIFs (PhPIFs). Expression levels of petunia *PIF*s in petals of 1 day postanthesis (1 dpa) flowers in the morning (10.00 h) and in the evening (19.00 h). Data are average normalized counts of the three biological repeats ± SEM, obtained from the RNA-seq data (Shor et al., 2023b). Significance of differences was calculated using Student's t-test, $*P \leq 0.05$.

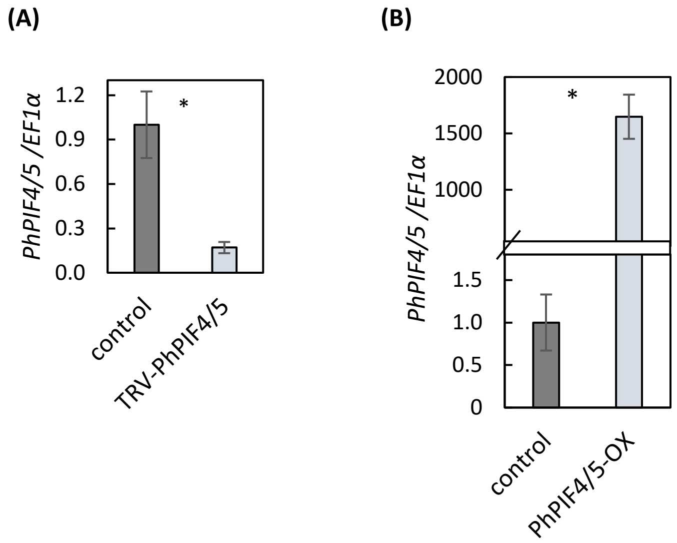

Supplementary Figure 2. Relative expression levels of *PhPIF4/5* in agroinfiltrated petunia petal regions. Petals were inoculated with *Agrobacterium*, carrying (A) TRV-PhPIF4/5 or TRV-CHS as a control; (B) 35S:*PhPIF4/5* (*PhPIF45–OX)* or *DsRED-OX* as a control. Samples were collected for RNA extraction 2 days after infiltration at 11.00 h. *EF1α* was used as an internal reference gene. The expression level of the target was normalized to that in the control. Data are means ± SEM, n = 4–6. Significance of differences between treatments was calculated using Student's t-test, *$P \leq 0.05$.

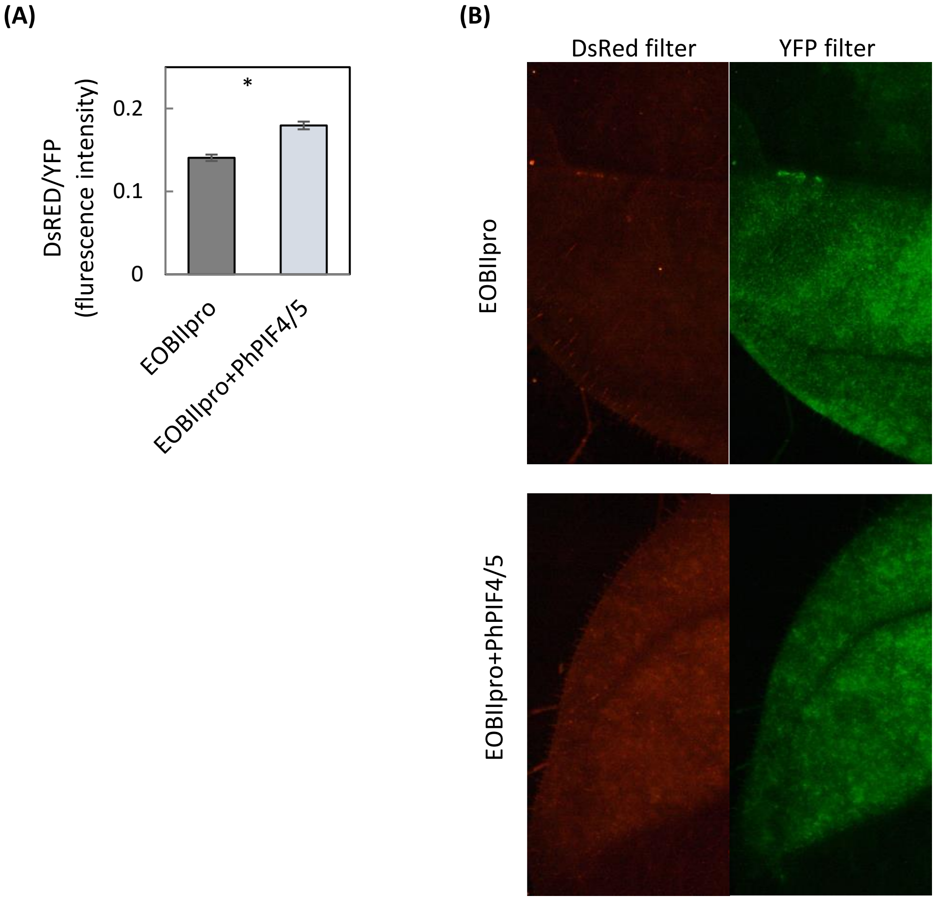

Supplementary Figure 3. *EOBII* promoter is activated by PhPIF4/5 in petunia leaves. Leaves were agroinfiltrated with binary vector carrying *DsRED* under the native *EOBII* promoter and *35S*:*PhPIF4/5* (*EOBII*pro+*PhPIF4/5*). As a control, binary vector without PhPIF4/5 (*EOBII*pro) was used. For normalization, petals were co-infiltrated with a vector carrying *35S*:*YFP*. (A) Relative DsRED levels, estimated by imaging as DsRED/YFP fluorescence signal ratio (n = 15). Data are means ± SEM. Significance of differences between treatments was calculated using Student's t-test, $*P \leq 0.05$. (B) Fluorescence images of leaves 4 days after infiltration.

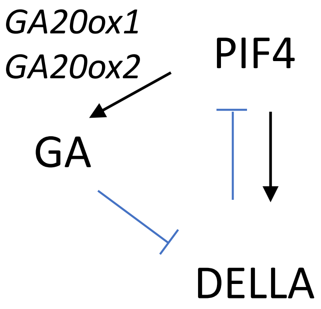

Supplementary Figure 4. A model showing PIF4–DELLA fine-tuning in the modulation of GA signaling in *Arabidopsis*. PIF4 together with PIF5 are required for activation of GA-biosynthesis genes *GA20ox1* and *GA20ox2*. GA leads DELLA proteins to degradation. DELLAs, in turn, negatively control the abundance of PIFs, while PIF4 activates expression of DELLA *RGA2*. Black lines indicate regulation at RNA level, blue lines – at protein level.

Supporting information

Table S1. Primers used for qRT-PCR analyses

	qRT-PCR primers (5'->3')	
Gene	**Forward primer**	**Reverse primer**
EF1α	TGAGATTCTGCGTGGATGAA	CCCATCAAGCAACTTGGACT
EOBI	TTCTCCATCTTCATACACTGGAAATA	GCTAGGCAGCTAGATTACTGATT
EOBII	CCATTGATCAGACCTACTCTCCCAC	GGACCAGATGTCTTCCATGCTCC
ODO1	ACCAACC`TACCAACCAACCA	ATGATGACCCCTCCAACAAG
EPSPS	GCGGAAAACCTTCCCTAACT	GCCCTTCTGAACTGAAATGG
ADT1	GATCGCGCAGTTCTACCAGTAG	AAGAGGCAGTGATGAACGGG
PAAS	TGTCGATGAAACCCAAGTGA	ACCACATTCCAGGCCATATC
PAL2	TGCTAATGGTGAACTTCATCCA	TGACATTCTTCTCACTTTCACCA
IGS	CCACGTCAAAAGAGTGAGCA	CCAGTGGTTTTCTCCCAAGA
BSMT1	GTGGTCGAAAAACCCGAATA	ATTAGCACCTCGACCACGAC
BPBT	TGTTGAAGGGTGATGCTCAA	GGATTTGGCATTTCAAACAAA
PhPIF4/5	ACAGTGGATCAGCACAGCAG	CAACTTGATTGCTGCCACAG
PhDELLA1	AGGTGGTCATGGCTATCGAG	CTTCTAGTTGTGGTGGGGCT
PhDELLA2	ACTTGGTTTTGTAAAGGTGGGG	GTGTATTGGGGAGTGGGGAA
RGA1 (At)	CCGGAGATTTTCACTGTGGT	AACTCGGTCAGGTCCATCAC
RGA2 (At)	CCGGAAACGCGATTTATCAGT	GTCGTCACCGTCGTTCCTAT
tubulin (At)	ACTCACTACCCCCAGCTTTG	GACCAGGGAACCTCAGACAG
DsRED	TCCCCGACTACAAGAAGCTGT	CCATGGTCTTCTTCTGCATCA
YFP	CTATATCATGGCCGACAAGCA	GGGTGTTCTGCTGGTAGTGGT
